# Treatment of Diphtheria

**Published:** 1878-06

**Authors:** 


Treatment of Diphtheria.—During the past season a very malignant epidemic of diphtheria prevailed in a region of Rhode Island of which Little Comfort is the centre. It attacked all ages, and was very fatal, notwithstanding the conscientious trial of all the usual remedies. Dr. White, of Little Comfort, finally hit upon the following remedy, the success of which was, in almost every case, prompt and efficient:

| | |
|---|---|
| Pulv. cinchona, . . . | 2 parts. |
| " capsici, . . . . | 2 parts. |
| " ipecac, . . . . | 1 part. |

M.—Sig. 5 to 10 grains every 2 hours.

Dr. W. subsequently made a tincture from the above powder, which was administered in doses of from 5 drops to a teaspoonful every two hours. Since last fall three barrels of this tincture have been sold.—*Hospital Gazette.*

